# New sites of localisation of *Pasteurella multocida* B:2 in buffalo surviving experimental haemorrhagic septicaemia

**DOI:** 10.1186/1746-6148-10-88

**Published:** 2014-04-11

**Authors:** Salleh Annas, Mohammad Zamri-Saad, Faez Firdaus Abdullah Jesse, Zakaria Zunita

**Affiliations:** 1 Research Centre for Ruminant Diseases, Faculty of Veterinary Medicine, Universiti Putra Malaysia, 43400 Serdang, Malaysia; 2 Department of Clinical Studies, Faculty of Veterinary Medicine, Universiti Putra Malaysia, 43400 Serdang, Malaysia; 3 Department of Veterinary Pathology and Microbiology, Faculty of Veterinary Medicine, Universiti Putra Malaysia, 43400 Serdang, Malaysia

**Keywords:** *Pasteurella multocida* B:2, Haemorrhagic septicaemia, Carrier, Respiratory, Gastrointestinal tract, Urinary tract

## Abstract

**Background:**

Haemorrhagic septicaemia (HS) is an acute septicaemic disease of buffalo and cattle caused by *Pasteurella multocida* B:2 and E:2. Field outbreaks of HS are known to result in localisation of bacteria in the tonsils of surviving buffalo, confirming that animals can become carriers and the role of respiratory tract in the transmission of the disease. This report describes additional sites of localisation of *P. multocida* B:2 in surviving buffalo following experimental induction of HS.

**Results:**

Following *P. multocida* B:2 infection, all calves in group 1 and one calf in group 2 that was allowed to commingle with infected calves from group 1 were euthanised within 48 h. *Pasteurella multocida* B:2 was detected from the nasal and rectal swab samples on days 5 and 6 from the remaining calves in group 2. The first injection of dexamethasone into the carrier animals resulted in reemergence in samples from the nose, rectum and vagina. However, subsequent dexamethasone injections failed to re-activate *P. multocida* B:2. When surviving carrier calves in group 2 were euthanised at the end of the experiment, *P. multocida* B:2 was detected in the lungs and various organs of the respiratory, gastrointestinal and urinary tracts.

**Conclusions:**

Commingling naive buffalo calves with calves acutely infected with *P. multocida* B:2 resulted in carriers among surviving buffalo. *Pasteurella* was found in various organs of the respiratory, gastrointestinal and urinary tracts, suggesting their role in the pathogenesis of HS.

## Background

Haemorrhagic septicaemia (HS) is an acute, fatal, septicaemic disease of cattle and buffalo caused by two specific serotypes of *Pasteurella multocida*: B:2 (Asian serotype) and E:2 (African serotype). HS has a wide distribution, especially in South East Asia and Africa [1]. On the Malaysian Peninsula, a total of 48 outbreaks of HS have occurred between 1994 and 2005, resulting in a total of 921 dead buffalo and 394 dead cattle [2]. A recent outbreak in India involving free-range cattle and buffalo resulted in 52 deaths, a mortality rate of 33.76% [3]. In ruminants, natural exposure to *P. multocida* usually results in rapid disease progression, with typical clinical signs of severe depression, pyrexia, submandibular oedema, dyspnoea, recumbency and death. Transmission is typically by carrier animals [4], which are animals that have survived a previous outbreak [5]. Previous studies on carriers were focused on the respiratory tract [5-8] following detection of *P. multocida* B:2 in the tonsil, nasopharynx and upper respiratory tract lymph nodes [5,9,10]. Both natural and experimentally induced stresses aggravate shedding and transmission of the bacterium [5,11].

One recent study has indicated gastrointestinal tract involvement in acute HS [12]. However, there have been no studies on the role of the gastrointestinal and urinary tracts in carriers and transmission of HS. This paper describes the experimental development of carrier buffalo and the role of gastrointestinal and urinary tracts of carrier buffalo in spreading HS.

## Results

### Clinical responses

All calves of group 1 were euthanised following development of advanced clinical signs; two were euthanised at 24 h post-infection, while the rest were euthanised at 48 h post-infection. Affected calves appeared dull and depressed, recumbent, pyrexic, anorexic and dyspnoeic, with nasal discharge and diarrhoea.

**Figure 1:**
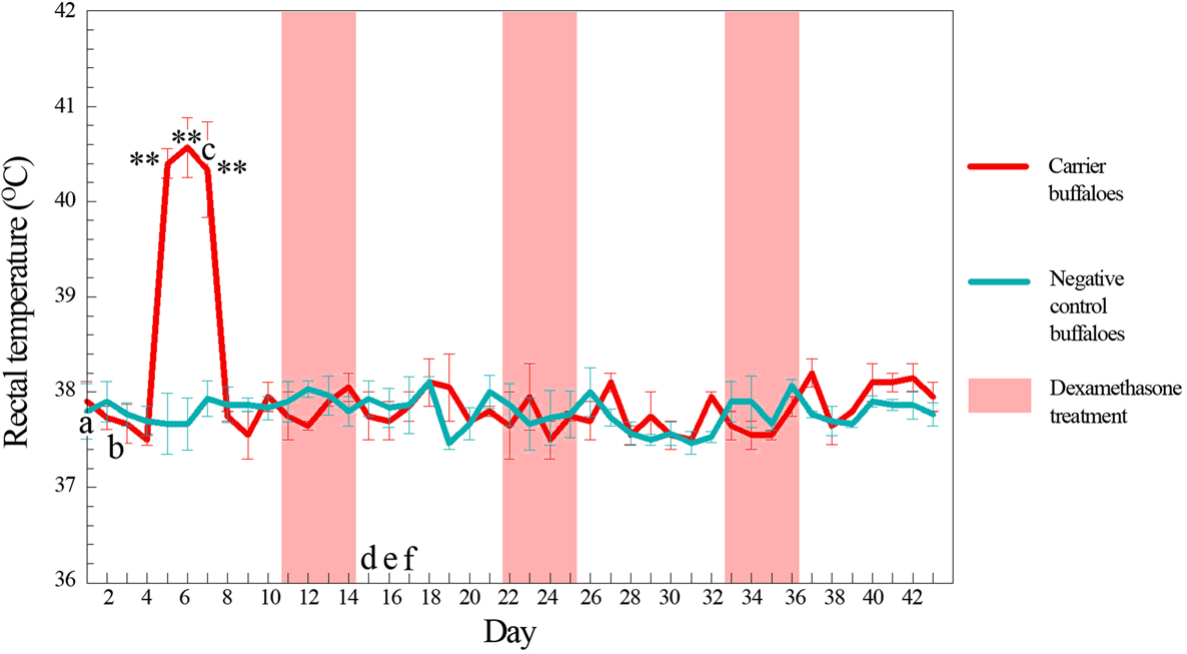
**Time line and mean rectal temperature: comparison between carrier animals and negative control animals.** **indicates a significant difference of p < 0.05 compared with the negative control group. a: Day 1: inoculation of *P. multocida* B:2 to group 1. b: Day 2: all animals in group 1 euthanised. c: Day 5 and 6: positive isolation made from nasal and rectal swabs from carrier animals in group 2 (2A, 2B, 2C). Day 7: 2D euthanised. d: Day 15: positive *P. multocida* B:2 from rectal swab of 2A. e: Day 16: positive *P. multocida* B:2 from nasal swabs of all carriers and rectal swab of 2B. f: Day 17: positive *P. multocida* B:2 from nasal swab of 2A as well as rectal and vaginal swabs of all carriers.

On days 5, 6 and 7 after being mixed with the calves from group 1, three calves (2A, 2B and 2C) of group 2 were transiently pyretic, with average body temperatures of 40.4°C (SEM = 0.15), 40.6°C (SEM = 0.32) and 40.3°C (SEM = 0.50), respectively (Figure 1). Another calf (2D) showed progressive pyrexia for more than 48 h and was euthanised because of severe clinical signs. The surviving calves in group 2 eventually became carriers. None of the calves in group 3 showed clinical signs.

### Gross pathology

In general, all calves of group 1 and one calf (2D) from group 2 that were euthanised following the development of severe clinical signs had classic lesions of acute HS, including severe congestion and haemorrhage of mucosal surfaces, subcutaneous tissues and respiratory, gastrointestinal and urinary tracts. Fibrin was deposited on the serosal surfaces of all thoracic and abdominal organs.

The surviving calves of group 2 had multifocal pulmonary congestion and pneumonia. Fibrin was deposited on the omentum, mesentery, peritoneum and serosal surfaces of the gastrointestinal tract. However, the mucosal surfaces of the gastrointestinal tract appeared normal apart from the rectum, which had mild multifocal haemorrhages.

### Isolation and identification of *P. multocida* B:2

*Pasteurella multocida* B:2 was successfully isolated from organ samples of all calves that were euthanised because of acute HS.

*Pasteurella multocida* B:2 was cultured from nasal and rectal swabs collected on days 5 and 6 from the surviving buffalo calves (2A, 2B, 2C) of group 2 on post-exposure days 5–7 and 15–17 (5–7 days after the administration of the first course of dexamethasone). *Pasteurella multocida* B:2 was also isolated from vaginal swabs of these calves on post-exposure days 15–17. Isolation was unsuccessful between days 7 and 14 and following the second and third courses of dexamethasone (Figure 1). At the end of the 43-day experiment, *P. multocida* B:2 was detectible via polymerase chain reaction (PCR) in the tonsils, lung, reticulum, ileum and ureter of calves 2A, 2B and 2C (Table 1).

**Table 1:** ***Pasteurella multocida *** **B:2 isolation, DNA detection and localisation in all samples from calves 2A, 2B and 2C**

**Tract**	**Organ**	***P. multocida*** **B:2 DNA (+/total)**	**Localisation**
Respiratory	Nasal	0/3	Serous glands.
	Tonsil	3/3	Tonsillar crypts with denuded epithelial cells.
		(Animal ID: 2A, 2B, 2C)	
	Epiglottis	0/3	Epithelium, serous glands.
	Trachea	0/3	Epithelial surface.
	Lungs	1/3	Bronchiolar exudates, alveolar space, alveolar sac surface, intracytoplasmic of alveolar macrophages.
		(Animal ID: 2A)	
Gastrointestinal	Liver	0/3	(negative)
	Rumen	0/3	Tip of mucosal surface.
	Reticulum	1/3	
		(Animal ID: 2B)	
	Omasum	0/3	
	Abomasum	0/3	
	Duodenum	0/3	
	Jejunum	0/3	
	Ileum	1/3	
		(Animal ID: 2C)	
	Caecum	0/3	
	Colon	0/3	
	Rectum	0/3	
	Mesenteric lymph nodes	0/3	(negative)
Urinary	Kidney	0/3	(negative)
	Ureter	1/3	Ureteral lumen, intracytoplasmic of denuded epithelial cells.
		(Animal ID: 2C)	
	Urinary bladder	0/2	Intracytoplasmic and surface of transitional cells.

### Immunoperoxidase examination

**Figure 2:**
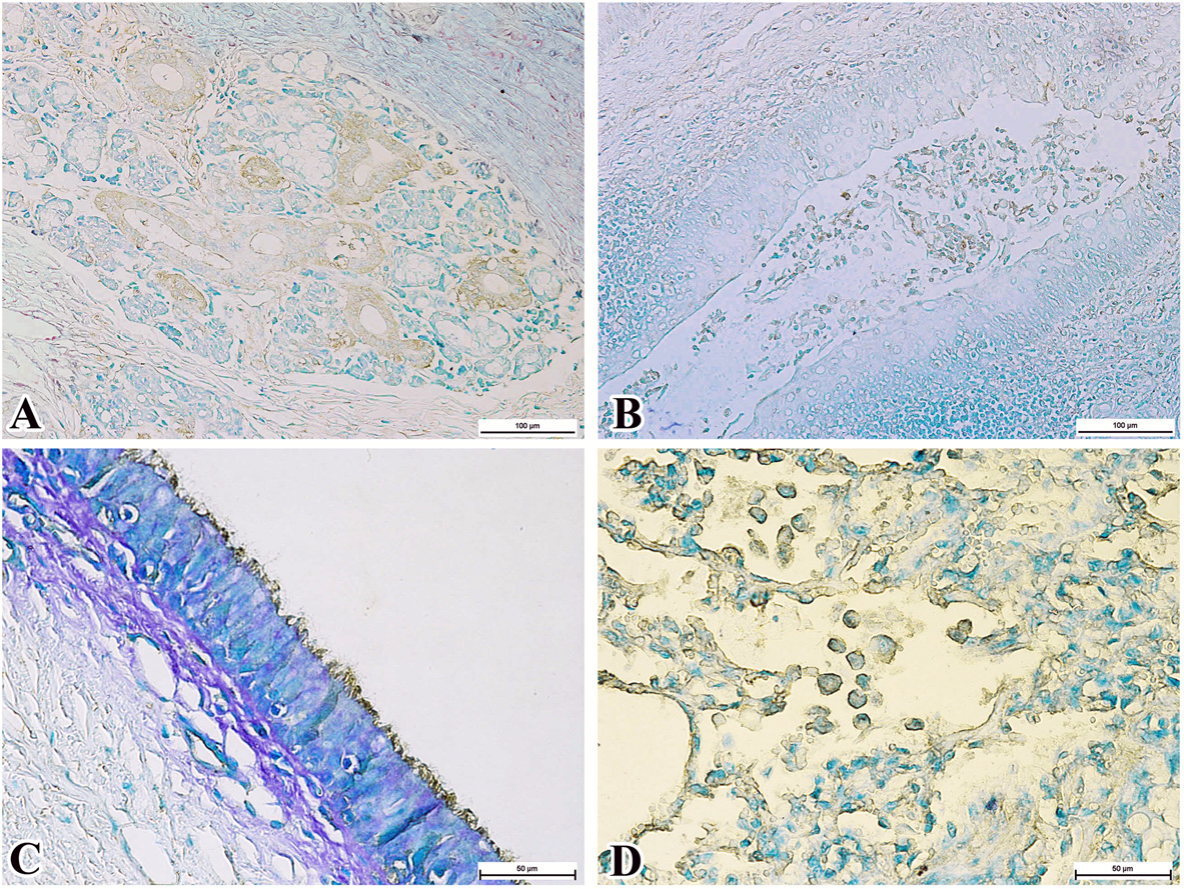
**Immunolocalisation of *P. multocida *B:2 in the respiratory tracts of carriers. A**. Serous glands in the nasal passage (bar = 100 μm). **B**. Tonsillar crypts mixed with cellular debris (bar = 100 μm). **C**. Ciliated tracheal epithelium (bar = 50 μm). **D**. Intracytoplasmic immunoreactivity in alveolar macrophages and on the surface of the alveolar sac (bar = 50 μm).

The respiratory, gastrointestinal and urinary tracts of all surviving group 2 calves had mild to moderate *P. multocida* B:2 immunoreactivity. Within the respiratory tract, immunoreactivity was noted in the nasal mucosa, tonsil, epiglottis, trachea and the lungs. In the nasal mucosa, the cells of nasal glands showed intracytoplasmic immunoreactivity (Figure 2A) while the tonsillar epithelium and tonsillar crypts had immunoreactivity amidst cellular debris (Figure 2B). Rare immunopositive bipolar rods were detected on the tonsillar epithelium and crypts.

Similar immunoreactivity was detected in the cells of serous glands, the epithelial cells of the epiglottis and on the epithelium of the trachea (Figure 2C). In the lungs, positive immunoreaction was observed on the surface of pneumocytes. Intracytoplasmic immunoreaction was observed in occasional infiltrating and degenerating alveolar macrophages (Figure 2D). Positive immunoreactivity was also detected in bronchiolar exudates that were mixed with desquamated bronchiolar epithelial cells.

**Figure 3:**
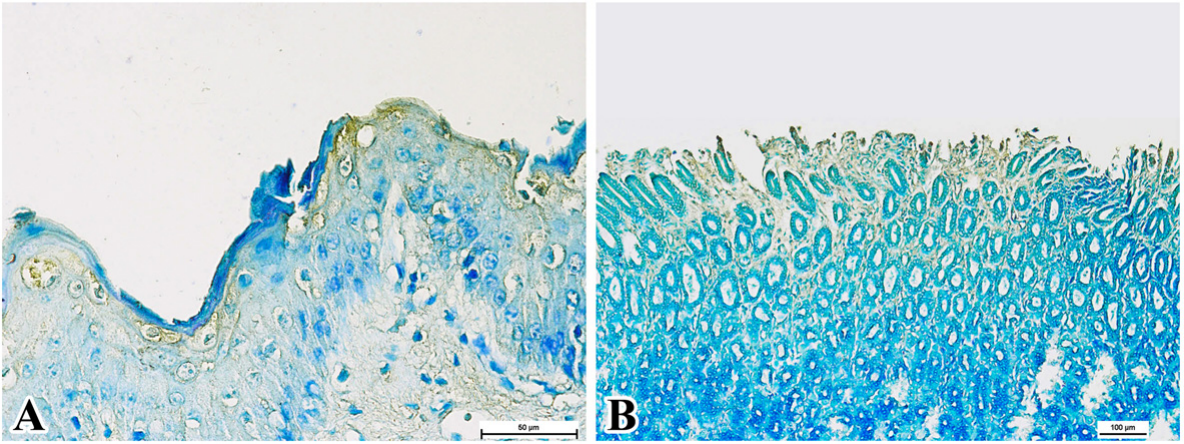
**Immunolocalisation of *P. multocida *B:2 in the gastrointestinal tract of carriers. A**. Keratinised mucosa of reticulum (bar = 50 μm). **B**. Abomasal mucosa (bar = 100 μm).

Within the gastrointestinal tract, all organs had immunoreaction except for liver and mesenteric lymph nodes. The rumen, reticulum and omasum had positive immunoreactivity beneath the keratin layer (Figure 3A), while the abomasum was immunoreactive at the tips of the mucosal surface (Figure 3B). Similar positive reactions were found at the tips of the mucosal surfaces in all sections of small and large intestines and within rare infiltrating macrophages.

**Figure 4:**
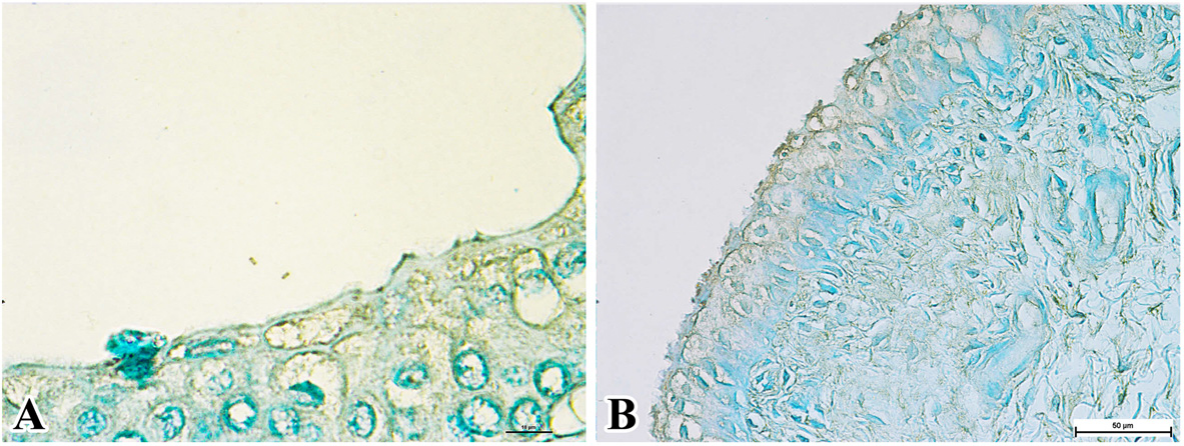
**Immunolocalisation of *P. multocida *B:2 in the urinary tract of carriers. A**. Ureteral epithelium. Note the presence of two immunopositive bipolar rods suggestive of *P. multocida* B:2 (bar = 10 μm). **B**. Urinary bladder wall (bar = 50 μm).

There was no immunoreactivity in any kidney samples. However, the ureter showed mild intracytoplasmic immunoreaction within the epithelial cells. Few immunopositive bipolar rods were detected in ureteral lumina (Figure 4A). Similar mild immunoreactions were observed in sections from the urinary bladder, particularly in the urothelial cells (Figure 4B).

**Figure 5:**
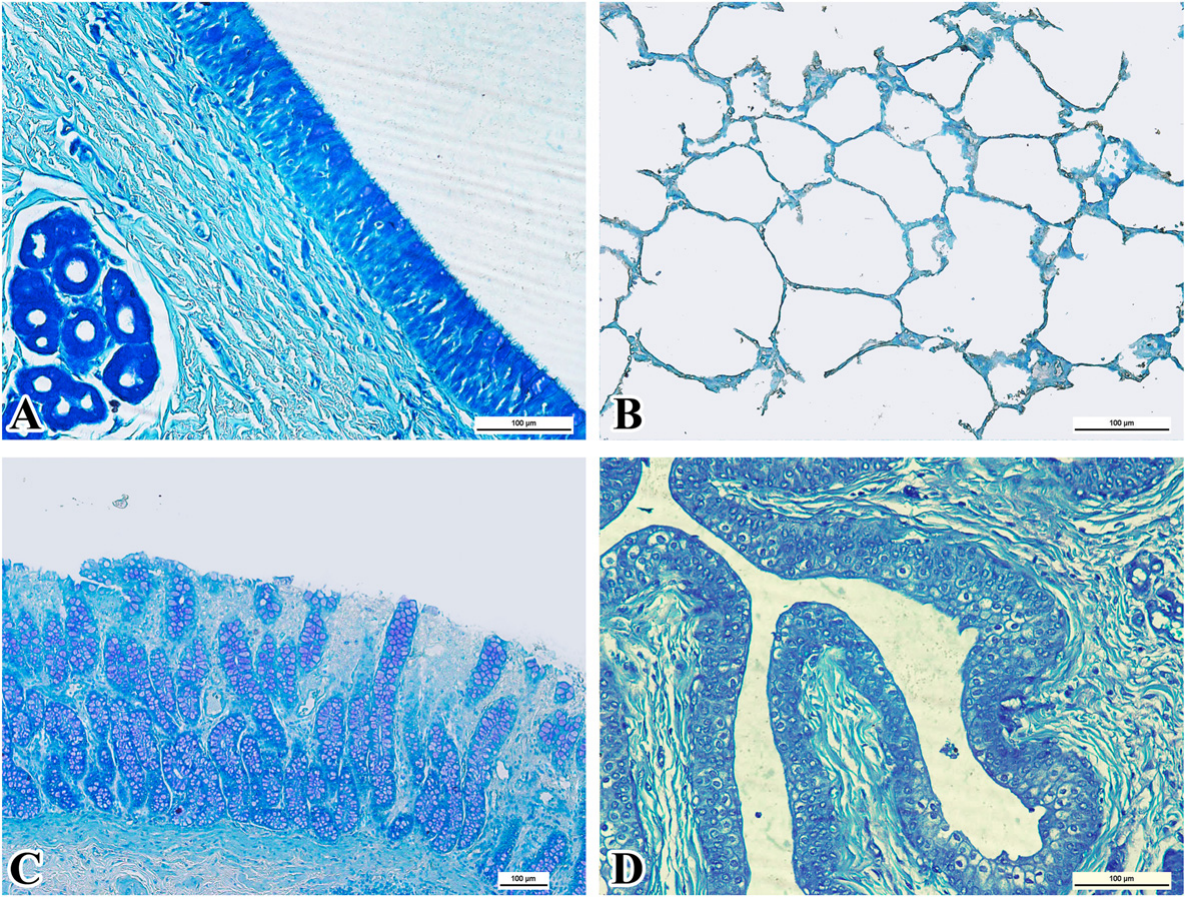
**Negative control samples. A**. Trachea (bar = 100 μm). **B**. Lungs (bar = 100 μm). **C**. Rectum (bar = 100 μm). **D**. Ureter (bar = 100 μm).

There was no immunoreactivity in any tissues from calves in group 3 (Figure 5).

## Discussion

In this experiment, carriers were detected among surviving buffalo that were allowed to comingle with the infected buffalo, simulating field outbreaks of HS. In addition to carriers, commingling buffalo can die of HS, as observed in calf 2D. Susceptible animals have been shown to rapidly succumb to HS following exposure to a high dose of *P. multocida* B:2, because of either a high growth rate within the host or the presence of other virulence factors that enable the agent to overcome the host’s defense mechanisms. Furthermore, poor development of specific and non-specific immunity of the host contributes to the development of acute HS [13]. The involvement and interactions between these factors are not fully understood [5]. If the host’s defense mechanisms prevail against the agent, the animals become carriers, as observed among the surviving buffalo calves of group 2 [11]. However, mixing naive animals with acutely infected animals to create carriers has the limitation of not being able to control the infection dose [5]. If the naive animals survive and become carriers, there may be individual differences in clinical signs, tissue tropism and disease course. It does have the advantage of more closely replicating field disease exposure.

Administration of courses of dexamethasone to carriers simulated stress and immunosuppression [12,14], leading to shedding and detection of *P. multocida* B:2 in the respiratory, gastrointestinal and urinary tracts. This confirms the involvement of the gastrointestinal and urinary tracts as potential transmission routes for HS by carriers following stress. This suggests a mechanism for outbreaks of HS that occurred during stressful monsoon seasons [4].

Immunoperoxidase staining localised *P. multocida* B:2 within the respiratory, gastrointestinal and urinary tracts of carriers. In HS, there are two types of carriers: active and latent. Active carriers actively shed bacteria and the agent may be cultured or detected during a post-mortem examination. Latent carriers do not shed bacteria but the agent can be cultured or demonstrated at low concentrations during post-mortem [4,5,15,16]. In this study, *P. multocida* B:2 was detected in the respiratory, gastrointestinal and urinary tracts at relatively low concentrations, which suggests these animals were latent carriers [17]. At the time of euthanasia, the agent could no longer be isolated, unlike previous studies [5,7,8] and was associated with mild immunoreactivity on the mucosal surfaces of the respiratory, gastrointestinal and urinary tracts. Demonstration of immunoreactive bipolar rods is consistent with infiltration of the tonsil, as suggested by previous studies [5,9,10,14,18]. However, this study localised *P. multocida* B:2 to the gastrointestinal and urinary tracts of carrier buffalo for the first time. While the tonsil may be the most important tissue reservoir in carriers, it is not the only organ of concern.

## Conclusions

Successful isolation of *P. multocida* B:2 from nasal, rectal and vaginal swabs suggests that buffalo that survive experimental outbreaks of HS become carriers. The organism is not only located in the respiratory tract as previously believed, but is also detectable in the gastrointestinal and urinary tracts, suggesting their involvement in the transmission of HS.

## Methods

### Animals

Twelve eight-month-old, clinically healthy, buffalo calves from a farm with no history of HS or vaccination against *P. multocida* were selected for the study. Calves were acclimatised for a period of 5 days and treated with anthelmintics. Nasal swabs were collected daily to ensure calves were free from *P. multocida* by culture and PCR [19]. Calves were fed cut grass supplemented with 400 g/calf/day of palm kernel-based pellets. Drinking water was available *ad libitum*. Access to veterinary care was available at all times and the well-being of the calves was assessed regularly.

### Inoculum preparation

Stock cultures of *P. multocida* B:2 isolated from a previous outbreak of HS were used to prepare the inoculum. Bacteria were cultured onto blood agar plates and incubated at 37°C for 24 h. Four colonies were inoculated into brain-heart infusion broth and incubated at 37°C with shaking at 150 revolution per minute (rpm) for 18 h. Bacteria were quantified via serial dilution, and an infective inoculum containing 1.0 × 10^5^ colony forming units (cfu)/mL was prepared [20].

### Experimental design

All experiments were approved by the Animal Care and Use Committee of Universiti Putra Malaysia (approval number 12R148).

Calves were divided into three equal groups. Group 1 served as the infected group, group 2 (consisting of calves 2A, 2B, 2C and 2D) as the commingling group and group 3 as the negative control group. All calves of group 1 were challenged subcutaneously at the shoulder with 5 mL of inoculum containing 1.0 × 10^5^ cfu/mL of *P. multocida* B:2 [21]. Calves of group 2 were not infected but were kept together and were allowed to commingle with the infected calves of group 1 [22]. Calves of group 3 were inoculated subcutaneously at the shoulder region with sterile phosphate-buffered saline and housed separately. Following inoculation, all calves were observed for clinical signs of HS and rectal temperatures were checked daily. Calves with advanced clinical signs were euthanised according to the guideline of the Ethics Committee, Universiti Putra Malaysia.

Deep nasal, rectal and vaginal swabs were collected daily from all animals, throughout the 43-day study period using sterile cotton swabs. Deep nasal swabs were obtained by cleaning the external nares with 70% alcohol, inserting the swabs 15 cm into the nostrils, and swabbing the nasal and nasopharyngeal mucosa by rotating the swabs several times. The rectal and vaginal swabs were obtained by cleaning the rectal and vaginal regions with 70% alcohol, inserting the swabs 7 cm into the rectum or vagina, and swabbing the vaginal and rectal mucosa by rotating the swabs several times. All swab samples were immediately transported to the laboratory in Cary Blaire medium.

Once a week had passed with no positive cultures, calves were injected intramuscularly with dexamethasone (Dexason; Troy Laboratories) for 3 consecutive days at the rate of 1 mg/kg to induce immunosuppression [12]. After the first set of injections, two additional series of dexamethasone injections followed at 7-day intervals.

After the final dexamethasone injection, all surviving calves in groups 2 and 3 were euthanised. Samples of nasal passage, tonsil, epiglottis, trachea, all eight lung lobes, liver, rumen, reticulum, omasum, abomasum, duodenum, jejunum, ileum, mesenteric lymph nodes, caecum, colon, rectum, kidney, ureter and urinary bladder were collected from all euthanised animals in 10% neutral buffered formalin.

Tissue samples from group 1 were subjected to culture and identification of *P. multocida* B:2. Tissue samples from the surviving buffalo of group 2 and negative control of group 3 were subjected to culture and identification of *P. multocida* B:2, histopathologic and immunohistochemical examination.

### Isolation and identification of *P. multocida*B:2

All nasal, rectal and vaginal swabs were cultured on blood agar plates incubated at 37°C for 18 h. Identification of *P. multocida* B:2 was based on morphological criteria [23] and PCR [19]. Tissue samples were homogenised and diluted before deoxyribonucleic acid (DNA) extraction (NucleoSpin® Tissue; Macherey-Nagel, Düren, Germany). Purified DNA was subjected to detection by PCR [19].

### Localisation of *P. multocida* B:2

Tissue samples were fixed in 10% buffered formalin for at least 24 h before being trimmed, embedded in paraffin and sectioned at 3 μm. Sections were then subjected to immunoperoxidase and methylene blue stainings. Sections were dewaxed by incubation at 60°C for 15 min and immersion into xylene. Rehydration was via successive immersion in absolute alcohol, 90% alcohol, 70% alcohol and 50% alcohol. Sections were then exposed to citrate buffer using a carousel microwave at 50 W for 15 min and then Dako REAL™ Peroxidase Blocking Solution at room temperature for 30 min. Samples were incubated with primary rabbit hyperimmuneserum (1:200) against *P. multocida* B:2 at 37°C for 1 h, and then incubated with 1:1000 diluted peroxidase conjugated antiserum (polyclonal goat anti-rabbit immunoglobulin G (IgG); Dako) at 37°C for 30 min. Finally, sections were incubated with 3,3′-diaminbenzidine (DAB) (Dako Liquid DAB + SubstrateChromogen System) at room temperature for 2 min, followed by washing in distilled water and counterstaining with methylene blue. Finally, all sections were examined under a light microscope.

Adjacent sections were dewaxed and stained with methylene blue for 30 s before being examined under light microscope to detect bipolar *P. multocida* B:2.

### Statistical analysis

The Student’s *t* test was used to compare rectal temperatures between the carriers and control calves.

## Abbreviations

HS: Haemorrhagic septicaemia; CFU: Colony-forming units; rpm: Revolutions per minute; DNA: Deoxyribonucleic acid; PCR: Polymerase chain reaction; IgG: Immunoglobulin G; DAB: 3,3′-diaminbenzidine.

## Competing interests

The authors declare that they have no competing interests.

## Authors’ contributions

AS was involved in experimental design, sampling, sample processing, data analysis and drafting of manuscript. MZS was involved in supervision, research conception and design, data analysis, interpretation and manuscript revision. FFAJ was involved in experimental design, data interpretation and manuscript revision. ZZ was involved in supervision of the experiments and manuscript revision. All authors read and approved the final manuscript.
